# Comprehensive Analysis of the *GiTCP* Gene Family and Its Expression Under UV-B Radiation in *Glycyrrhiza inflata* Bat

**DOI:** 10.3390/ijms26010159

**Published:** 2024-12-27

**Authors:** Ziliang Liu, Jiaang Zhao, Ying Xiao, Caijuan Li, Rong Miao, Sijin Chen, Dan Zhang, Xiangyan Zhou, Mengfei Li

**Affiliations:** 1 College of Life Science and Technology, Gansu Agricultural University, Lanzhou 730070, China; 18419068256@163.com (Z.L.); changge0500@163.com (J.Z.); 15095576858@163.com (Y.X.); m17899316694@163.com (C.L.); m15379014783@163.com (R.M.); chenjj@gsau.edu.cn (S.C.); 2 State Key Laboratory of Aridland Crop Science, Gansu Agricultural University, Lanzhou 730070, China; zhangdan@gsau.edu.cn; 3 Agronomy College, Gansu Agricultural University, Lanzhou 730070, China

**Keywords:** *Glycyrrhiza inflata*, TCP transcription factors, transcriptomics, gene expression, subcellular localization

## Abstract

TCP is a plant-specific transcription factor that plays an important role in plant growth and development. In this study, we used bioinformatics to identify the entire genome of the *TCP* gene family in *Glycyrrhiza inflata* Bat, and we analyzed the expression characteristics of *GiTCP* genes under UV-B radiation using qRT-PCR. The results were as follows: (1) 24 members of the *TCP* gene family were identified in *G. inflata*, evenly distributed on its 24 chromosomes. (2) The *GiTCP* genes contained 0–4 introns and 0–5 exons. (3) The *GiTCP* genes were phylogenetically divided into three subfamilies—PCF, CIN, and CYC/TB1, with 14, 9, and 1 GiTCP proteins, respectively. (4) A covariance analysis showed that two pairs of *GiTCP* genes underwent a fragmentary duplication event. (5) A cis-element analysis showed that the cis-responsive elements of the *GiTCP* genes’ promoter regions were mainly comprised of light-responsive, stress-responsive, hormone-regulated, growth and development, and metabolic-regulated elements. (6) A protein network interaction analysis revealed a total of 14 functional molecules of TCPs and 8 potential interacting proteins directly related to GiTCP proteins. (7) GO annotation showed that the *GiTCP* genes were mainly enriched in BP, CC, and MF groups and had corresponding functions. (8) RNA-seq and qRT-PCR further indicated that *GiTCP3*, *6*, *7*, *8*, *12*, *14*, *17*, *23,* and *24* were up- or down-regulated in *G. inflata* after UV-B radiation, demonstrating that these genes responded to UV-B radiation in *G. inflata*. (9) Subcellular localization analysis showed that the GiTCP8 protein was localized in the nucleus. The results of this study provide a basis for further exploration of the function of the *GiTCP* gene family in the growth and development of *G. inflata*.

## 1. Introduction

Transcription factors (TFs) are a class of proteins with specialized structures and regulatory functions that play important roles in plant development and response to the external environment by activating or repressing gene transcription [1]. The *TCP* (teosinte branched 1/cincinnata/proliferating cell factor) gene family is a unique class of plant TFs gene. It consists of the *TB1* (teosinte branched 1) gene in maize (*Zea mays* L.), the *CYC* (Cycloidea) gene in *Anthurium majus*, and the *PCF1* and *PCF2* (PROLIFERATING CELL FACTORS 1 and 2) genes in rice (*Oryza sativa*) [2,3,4]. The N-terminus of the TCP protein, a transcription factor, has a highly conserved Helix–Loop-Helix (bHLH) domain, also known as the TCP structural domain, which has the function of binding DNA. The *TCP* gene family is divided into two subclasses, Class I and Class II: Class I is dominated by the *PCF* gene family, while Class II has a large diversity of members and is subdivided into two subfamilies, CIN and CYC/TB1 [5]. The former usually contains the glutamic acid–cysteine glutamic acid stretch (ECE) domain and an arginine-rich domain (R), while the latter usually has microRNA binding sites.

TCP proteins play important regulatory roles in plant growth, development, and response to biotic and abiotic stresses [6]. Research has shown that TCP transcription factors are involved in plant cell signaling and are associated with the biosynthesis of salicylic acid, ethylene, and abscisic acid [7], and they are also closely related to plant organogenesis, circadian rhythms, calcium homeostasis, and the regulation of sugar transport [8]. Additionally, *GmTCP* has been identified as a key candidate gene for the soybean nodule phenotype in response to nitrogen concentration [9].

Over the past few years, the *TCP* gene family has been identified and analyzed in many plants. A total of 40 *MsTCP* genes were identified in *Medicago sativa* L., of which *MsTCP23*, *MsTCP27*, *MsTCP29*, and *MsTCP33* demonstrated up-regulated expression under 15% PEG-6000 treatment [10]. There are a total of 48 *MaTCP* genes identified in bananas, and *MaPCF1*, *MaPCF5*, and *MaPCF21* were up-regulated under low-temperature stress, salt stress, and osmotic stresses [11]. A total of 60 TCP family members were identified in *Panax ginseng*. qRT-PCR analysis showed that *PgTCP20*, *PgTCP21*, *PgTCP22*, and *PgTCP23* were expressed in ginseng primary roots, baleen roots, rhizomes, stems, and leaves, while *PgTCP24* was not expressed in stems but was highly expressed in roots and rhizomes. The tissue-specific expression of these genes in ginseng showed their close relationship with growth and development [12]. Zhang et al. identified 23 *TCP* family members in *Andrographis paniculata*, and the expression levels of most *TCP* genes were higher in leaves and stems than in roots. Additionally, they found that under UV-B radiation, the expression levels of all *ApTCP* genes were down-regulated, except for those of *ApTCP2*, *ApTCP5*, *ApTCP6*, *ApTCP15*, *ApTCP18,* and *ApTCP23,* which were up-regulated. In addition, *ApTCP8*, *ApTCP9*, *ApTCP13,* and *ApTCP17* were not expressed [13]. A total of 50 TCPs were identified in three *Dendrobium* species, and transcriptomic analyses and qRT-PCR results indicated that *DchTCP2* and *DchTCP13* had significant effects on organ development. In addition, changes in the expression level of *DchTCP4* indicated its important role in the phenotypic variation in floral organs [14]. Although the *TCP* family has been extensively studied in various plant species, the *TCP* gene family in licorice has not been reported.

The Chinese medicinal herb licorice refers to the dried roots and rhizomes of the perennial herbaceous plants of the genus *Glycyrrhiza,* such as *G. uralensis* Fisch, *G. glabra* L., and *G. inflata* Bat. They are commonly used in clinical practice. Because of their ability to harmonize all medicines and detoxify all poisons, they are known as the “national old man” [15]. Secondary metabolites are the main medicinal components of licorice, and their contents are closely related to the environment. Studies have shown that moderate drought stress can promote the accumulation of active ingredients in licorice [16]. It was found that the relative content of glycyrrhizic acid and glycyrrhizin increased notably under salt stress by inhibiting primary metabolism and promoting secondary metabolism [17]. Appropriate concentrations of GA_3_ and MeJA can significantly promote the accumulation of active substances in licorice and the expression of genes related to their biosynthetic pathway [18,19]. At present, cultivated licorice is the main supply source of commercial licorice. The quality of cultivated licorice has become one of the main factors restricting its development, and improving its quality is currently a hot topic in licorice research. In recent years, functional genomics studies of licorice have also gained attention. The sequencing and assembly of the whole genome of licorice have been completed, providing a data basis for identifying gene families and studying gene functions at the whole-genome level [20]. Regarding transcription factor function studies, key genes involved in the synthesis of secondary metabolites have been unearthed through the identification of the *bZIP* and *WRKY* gene families in licorice [21,22]. Abiotic stresses due to environmental factors and cultivation conditions have become important factors affecting the variable quality of licorice.

Therefore, in this study, we comprehensively identified and analyzed the *GiTCP* gene family using bioinformatics, and we analyzed the expression levels of the *GiTCP* genes under UV-B radiation using transcriptomics and qRT-PCR, thereby laying the foundation for further research on the function of *GiTCP* genes.

## 2. Results

### 2.1. Identification and Physicochemical Properties of Members of the GiTCP Gene Family of G. inflata

Based on conserved structural domains, 24 members of the TCP family in *G. inflata* were identified from the transcriptomic data (PRJNA1086199), and they were sequentially named *GiTCP1~24* based on their chromosomal locations (Table 1). The physicochemical properties of the licorice *TCP* genes varied widely, with amino acid lengths ranging from 209 to 524. The molecular weight (MW) ranged from 22.11 to 57.43 kD. The isoelectric point (pI) ranged from 6.15 to 9.72, and acidic and basic proteins were evenly distributed. The instability indices ranged from 41.64 to 70.02, and they were greater than 40 for all family members, indicating that the *GiTCP* family was unstable. The fat index ranged from 46.27 to 72.65, with *GiTCP4* being the smallest and *GiTCP23* being the largest one. As the predicted total mean hydrophobicity (GRAVY) of GiTCP proteins ranged from 1.098 to 0.266, all of which were negative, it could be inferred that they were hydrophilic proteins. Subcellular localization predictions showed that all *GiTCP* genes localized to the nucleus, except for *GiTCP7~9*, *GiTCP11~17*, and *GiTCP20~24*, which also localized to the cytoplasm. In addition, *GiTCP2*, *GiTCP11*, *GiTCP12*, *GiTCP13*, *GiTCP16*, *GiTCP17*, *GiTCP18*, *GiTCP21*, *GiTCP22,* and *GiTCP24* localized to the chloroplast.

### 2.2. Phylogenetic Construction, Motif, Domain, and Gene Structure Analysis of the GiTCP Gene Family in G. inflata

**Figure 1:**
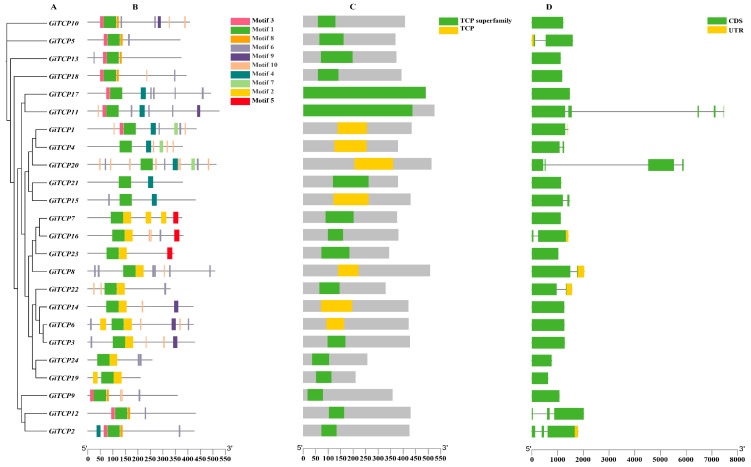
Phylogenetic relationship (**A**), conserved motifs (**B**), domains (**C**), and structures (**D**) of *GiTCP* genes.

Evolutionary trees were constructed individually for the members of the licorice *GiTCP* gene family (Figure 1A) and predicted using TBtools software (TBtools 2.007), https://tbtools.cowtransfer.com/s/0a9cbf41b47b4a (accessed on 11 January 2024). Ten conserved motifs were identified in the *GiTCP* gene family (Figure 1B). Analyzed in conjunction with the conserved structural domains, motif 1 had a complete sequence of TCP-conserved structural domains, and all 24 members of the *GiTCP* genes family were found to contain TCP structural domains (Figure 1C). The number of motifs in each member varied greatly: there was a minimum of two motifs, which appeared in the protein sequence of *GiTCP19* as a combination of motifs 1 and 2 and in that of *GiTCP21* as a combination of motifs 1 and 4, and there was a maximum of six motifs, with the number of motifs in *GiTCP1* and *GiTCP11* being the same, but *GiTCP1* contained motif 7 and *GiTCP11* contained motif 9. This study also found that some motifs appeared with certain regularity; for example, motif 2 was always associated with motif 9. The number of motifs in *GiTCP1* and *GiTCP11* was the same, while *GiTCP1* contained motif 7, and *GiTCP11* contained motif 9. In this study, we also found that motif 2 was always adjacent to and appeared after motif 1, and motif 3 was always adjacent to and appeared before motif 1. Thus, it was hypothesized that members containing the features of this sequence belong to the same subfamily. In order to further understand the evolutionary features of the *TCP* gene structure of licorice, we analyzed the distribution of the exons and introns of the *GiTCP* gene. The results showed that *GiTCP3*, *GiTCP6*, *GiTCP7*, *GiTCP9*, *GiTCP10*, *GiTCP13*, *GiTCP14*, *GiTCP17*, *GiTCP18*, *GiTCP19*, *GiTCP21*, *GiTCP23*, and *GiTCP24* had no intron structure; *GiTCP1*, *GiTCP4*, *GiTCP5*, *GiTCP8*, *GiTCP15*, *GiTCP16*, and *GiTCP22* contained one intron; *GiTCP2* and *GiTCP* contained two introns; *GiTCP20* contained three introns; and *GiTCP11* contained four introns (Figure 1D).

### 2.3. Distribution of GiTCP Genes on Chromosomes and Predictive Analysis of Secondary Structure in G. inflata

**Figure 2:**
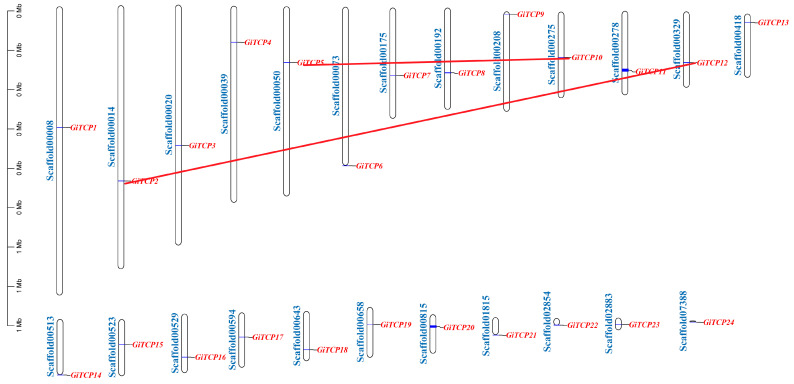
Chromosomal mapping analysis of the *GiTCP* gene family in *G. inflata*. Red lines represent the syntenic relationships of the *GiTCP* genes.

A chromosomal localization analysis revealed that the 24 *GiTCP* genes were evenly distributed on 24 chromosome scaffolds (Figure 2). A covariance analysis of the *GiTCP* genes family using MCScanX showed that two pairs of genes were segmental duplicates, *GiTCP2–GiTCP12* and *GiTCP5–GiTCP10*.

There are four main types of secondary structures of proteins, namely, α-helix, β-fold, β-rotor, and irregular curl. Usually, due to the relatively high molecular weight of proteins, different peptides of a protein molecule may have different secondary structures [23]. The prediction of the secondary structures of the 24 GiTCP proteins (Table 2) showed that they were mainly α-helical and irregularly coiled, with a smaller proportion of β-turned corner structures. Among them, GiTCP7 had the largest proportion of irregularly coiled structures, accounting for 71.02%. The proportion of its α-helical structures was 12.53%.

### 2.4. Phylogenetic Tree Analysis of TCP Genes

A phylogenetic tree was constructed using MEGA11 analysis software (MEGA 11.0.13) by performing 1000 repetitive searches for 24 GiTCP, 14 EuTCP [24], 24 AtTCP [25], and 40 MsTCP [10] proteins.

**Figure 3:**
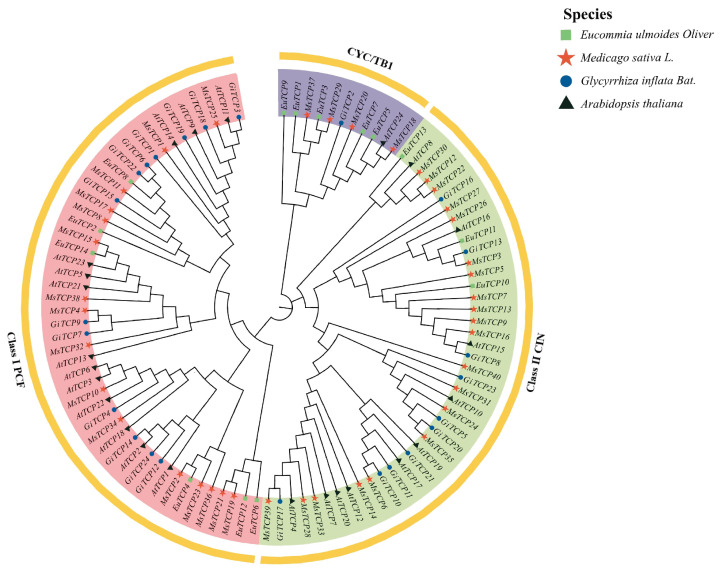
Phylogenetic tree analysis of the *TCP* gene family in *G. inflata* Bat, *Eucommia ulmoides* Oliver, *Medicago sativa* L., and *Arabidopsis thaliana*.

As shown in Figure 3, the 102 TCP proteins were categorized into two large subfamilies, Class I and Class II, of which Class I is also known as the PCF subfamily, and Class II is further divided into the CYC/TB1 and CIN subfamilies. The PCF subfamily contained the highest number of TCP members, with 48 TCP proteins, 14 GiTCPs, 5 EuTCPs, 13 AtTCPs, and 16 MsTCPs, followed by the CIN subfamily, which consisted of 43 TCP proteins, and the CYC/TB1 subfamily, which had the lowest number of proteins, containing only 11 TCP proteins, 1 GiTCP, 5 EuTCPs, 1 AtTCP, and 4 MsTCPs. Both licorice and alfalfa belong to the legume family and are closely related compared to the other two species.

### 2.5. Cis-Acting Element Analysis of GiTCP Genes in G. inflata

**Figure 4:**
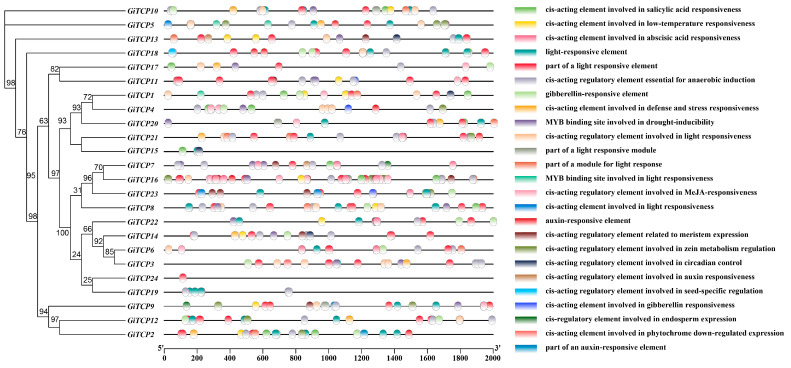
Analysis of cis-elements in the promoter region of *GiTCP* genes.

Cis-acting regulatory elements act as molecular switches and are closely associated with the regulation of gene expression under biotic and abiotic stresses [26]. To further understand the role of *GiTCP* genes in growth, development, and their response to environmental stresses, we utilized Plant CARE and TBtools to identify cis-regulatory elements within the 2000 bp promoter fragment located upstream of their start codons. The results showed that most of the promoters in the *GiTCP* genes had 24 original components related to light response, stress response, hormone regulation, growth and development, and metabolic regulation (Figure 4), with the largest number of regulatory elements being related to light response. They also had a considerable number of elements related to hormone response, such as growth hormone, gibberellin, jasmonic acid methyl ester, salicylic acid, and abscisic acid. In addition, there were a number of elements related to environmental stresses, such as low temperature, drought, defense, and stress response, as well as a small number of growth- and development-related elements, such as meristematic tissues, circadian rhythms, and endosperm expression. The largest number of light-responsive promoters was eight. Except for *GiTCP4*, light-responsive elements were found in all other *GiTCP* promoters, suggesting that *GiTCP* also has an important function in the response to light in *G. inflata*.

### 2.6. Protein Interaction Network Analysis of GiTCPs

**Figure 5:**
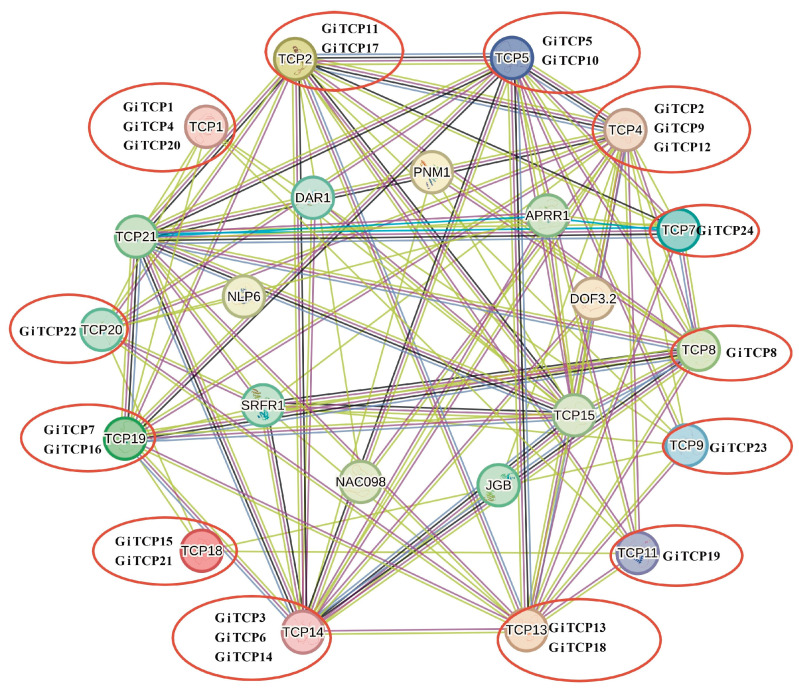
GiTCP protein–protein clustering interaction network diagram. (The red circles and spheres indicate licorice TCP proteins and Arabidopsis TCP proteins with which it interacts, respectively.)

Protein network interactions play multiple roles in plant growth and development. For example, in certain signaling pathways, different members of a gene family collaborate with each other to regulate the expression and activity of downstream target molecules, thereby affecting biological processes, such as cell growth, proliferation, and differentiation. In addition, protein network interactions can be involved in the regulation of important biological processes in plants, such as response to adversity, photosynthesis, plastid wall separation, and reproduction. To clarify the function of GiTCP proteins, this study used the model plant Arabidopsis to predict potential interacting proteins related to GiTCP protein function (Figure 5). NAC098 interacts with AtTCP4, suggesting that AtTCP4 (GiTCP2, GiTCP9, and GiTCP12) has a similar function. NAC098, a transcriptional activator of STM and KNAT6, is involved in the molecular mechanisms and regulates shoot apical meristem (SAM) formation during embryogenesis and organ segregation. It is also involved in the initiation of axillary meristems, the separation of meristems from the main stem, and the regulation of leaf sequencing throughout plant development and appears to be an inhibitor of cell division [27,28]. APRR1 controls the photoperiodic bloom response. The expression of several members of the APRR protein family, such as APRR9, APRR7, APRR5, APRR3, and APPR1, is controlled by circadian rhythms [29,30]. APRR1 interacts with AtTCP7 (GiTCP24), TCP14 (GiTCP3, GiTCP6, and GiTCP14), AtTCP15, and AtTCP21, suggesting that they have similar functions. The Dof3.2 transcription factor negatively affects seed germination and regulates a set of abscisic acid-related genes [31]. DAR1, together with DA1 and DAR2, regulates internal replication during leaf development. DA1, together with DAR2, regulates the protein stability of the transcription factors AtTCP14 and AtTCP15, which repress internal replication by directly regulating the expression of cell cycle genes [32]. AtTCP8 interacts with PNM1, an RNA-binding protein that functions in both the mitochondria and the nucleus. In the mitochondria, it is associated with multimerization and plays a role in translation, and it is involved in regulating the expression of its own genes in the nucleus [33]. SRFR1 interacts with AtTCP8, AtTCP14, AtTCP15, AtTCP20, and AtTCP21, localized proteins with which it interacts on the microsomal membrane [34].

### 2.7. GO Functional Annotation of GiTCP Genes in G. inflata

**Figure 6:**
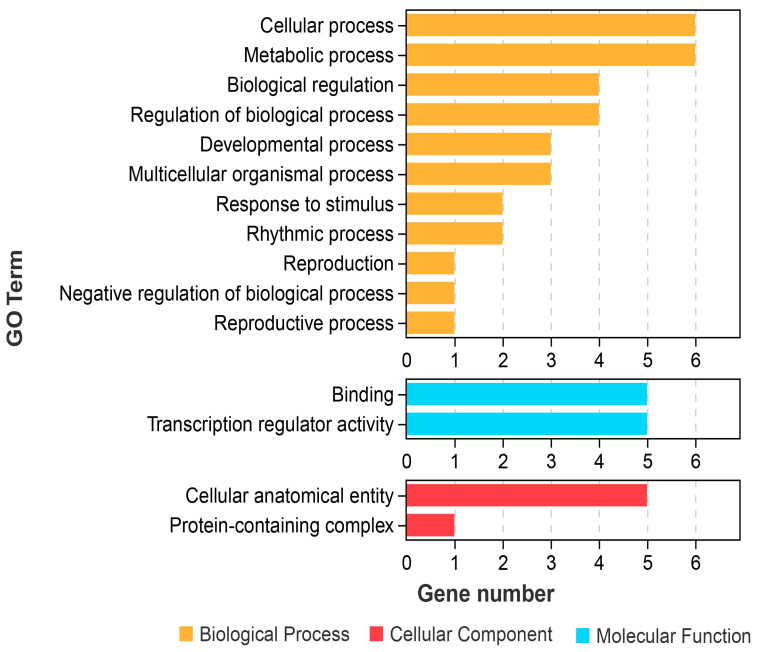
GO annotation of *GiTCP* genes in *G. inflata*.

Gene Ontology (GO) annotation of *GiTCP* genes was performed, as shown in Figure 6 and Table 3. The *GiTCP* genes were annotated, and only *GiTCP1*, *GiTCP14*, *GiTCP16*, *GiTCP19*, *GiTCP23,* and *GiTCP24* were found to be functional. They were categorized into 15 functional groups, such as “Biological Process (BP)”, “Cell Component (CC)”, and “Molecular Function (MF)”. In terms of the BP group, the main processes were reproduction (GO:0000003), metabolic process (GO:0008152), cellular process (GO:0009987), reproductive process (GO:0022414), multicellular organism process (GO:0032501), developmental process (GO:0032502), rhythmic process, (GO:0048511), negative regulation of biological process (GO:0048519), regulation of biological process (GO:0050789), response to stimulus (GO:0050896), and biological regulation (GO:0065007). In terms of the MF group, the main functions were binding (GO:0005488) and transcription regulator activity (GO:0140110).

### 2.8. Effect of UV-B Radiation on G. inflata

**Figure 7:**
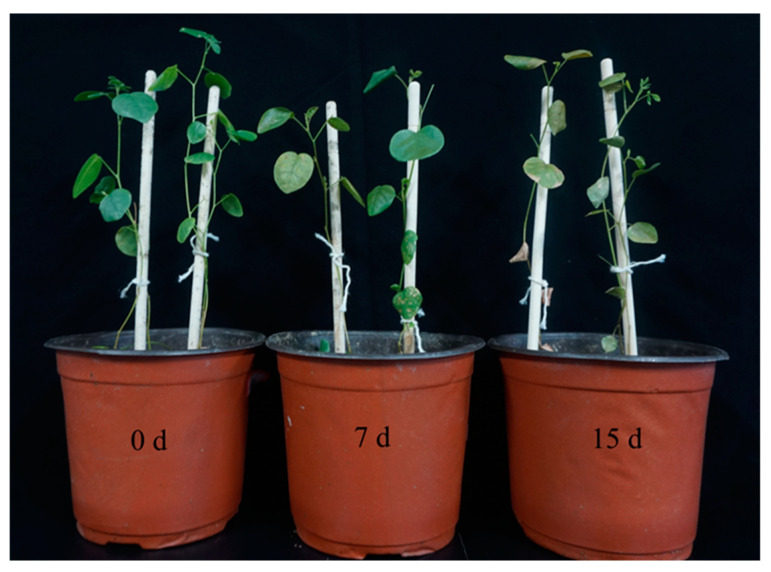
Changes in potted *G. inflata* seedlings cultured for 90 d with different time treatments (7 d, 15 d) under UV-B radiation (214 μw·m^−2^) and control (0 d) conditions.

In order to investigate the effect of UV-B radiation on *G. inflata*, an experiment was conducted to observe the phenotypic changes in potted licorice seedlings treated for different durations (0 d, 7 d, and 15 d). The results showed that licorice leaves exhibited no significant wilting or drying after 7 d of UV-B radiation. However, after 15 d, they began to curl and wilt (Figure 7).

### 2.9. Subcellular Localization Analysis

**Figure 8:**
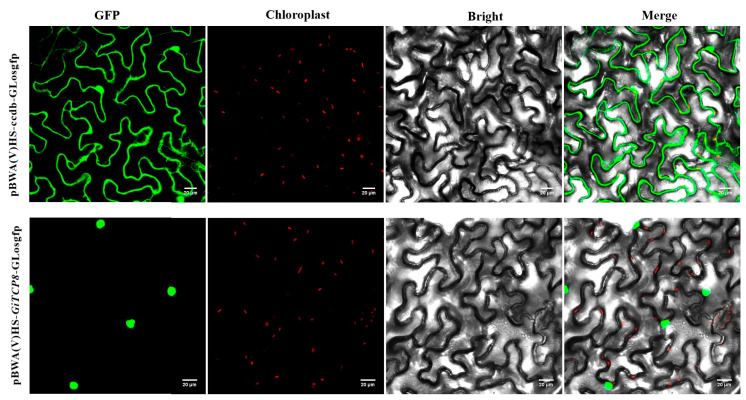
Subcellular localization of GiTCP8.

To further investigate the role of GiTCP in response to UV-B radiation in *G. inflata*, a highly expressed GiTCP8 protein was selected for in-depth functional studies. The pBWA(V)HS-*GiTCP8*-GLosgfp fusion protein was transiently expressed in tobacco leaves, and its subcellular localization revealed a strong fluorescent signal in the nucleus (Figure 8), suggesting that the GiTCP8 protein was localized in the nucleus. This observation was consistent with the results predicted by bioinformatics methods.

### 2.10. Expression Pattern Analysis of GiTCP Genes in Licorice Under UV-B Radiation

**Figure 9:**
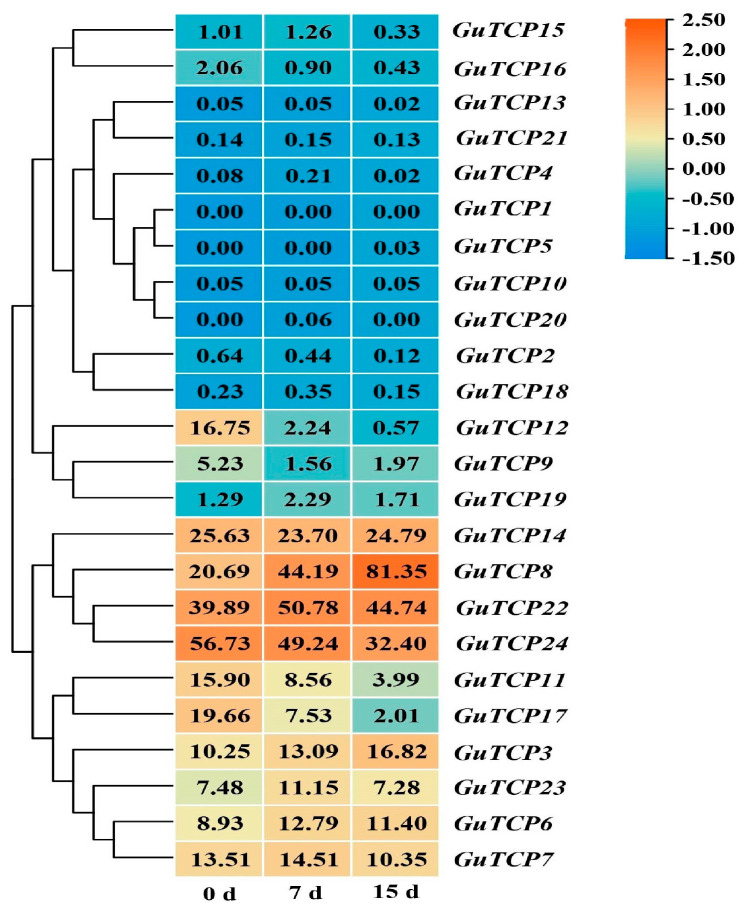
Expression of *GiTCP* genes based on transcriptomic analyses.

To investigate the expression patterns of the licorice TCP genes under UV-B radiation, an expression profile heatmap was plotted based on the FPKM values of raw RNA-seq data (Table S1). The results showed that *GiTCP8*, *GiTCP22,* and *GiTCP24* were highly expressed in the roots of licorice; *GiTCP1*, *GiTCP2*, *GiTCP4*, *GiTCP5*, *GiTCP10*, *GiTCP13*, *GiTCP18*, *GiTCP20,* and *GiTCP21* were barely expressed in the roots; and the other *GiTCP* genes showed a low expression. The expression of *GiTCP3* and *GiTCP8* increased with increasing radiation time in licorice. *GiTCP11*, *GiTCP12*, *GiTCP16*, *GiTCP17,* and *GiTCP24* were down-regulated in licorice with the increase in radiation time, and the expression of *GiTCP6*, *GiTCP7*, *GiTCP22,* and *GiTCP23* increased and then decreased with the increase in stress time in licorice. *GiTCP14* showed a decrease and then an increase in expression with increasing radiation time (Figure 9).

**Figure 10:**
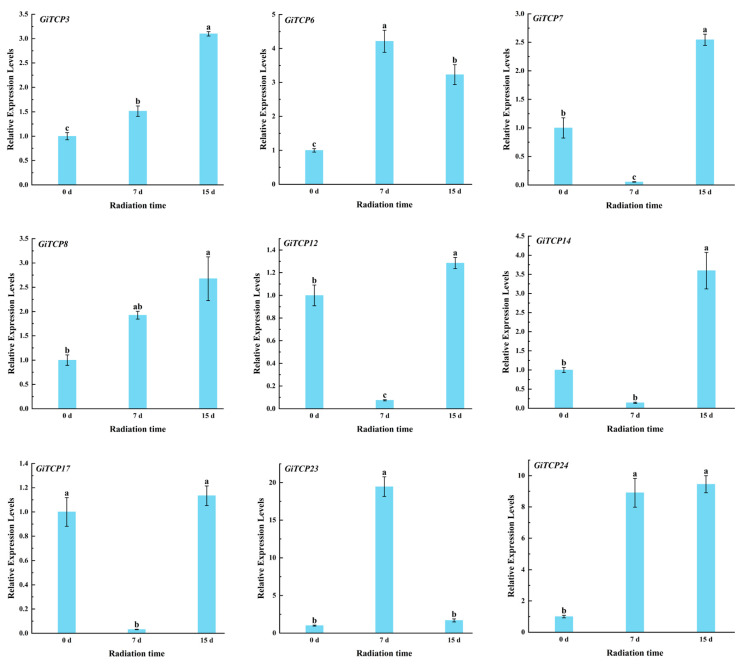
Expression patterns of the *GiTCP* gene family in response to UV-B radiation The character at the top of the error bar represents standard errors among three replicates, and different letters indicate significant differences among treatments (*p* < 0.05).

Nine *GiTCP* genes were selected for a qRT-PCR analysis to validate the results (Figure 10, Table S2), which showed that, under UV-B radiation, *GiTCP3*, *GiTCP8,* and *GiTCP24* were significantly up-regulated in licorice compared to the control (0 d). The relative expression of *GiTCP6* and *GiTCP23* peaked at 7 d of treatment. *GiTCP7*, *GiTCP12*, *GiTCP14*, and *GiTCP17* showed the lowest expression under 7 d of treatment compared to those under 0 d and 15 d radiation. Thus, the transcriptomic results and qRT-PCR results were almost identical, which indicates that the transcriptomic results of this study are reliable.

## 3. Discussion

The *TCP* gene family is a class of plant-specific transcription factors that play key roles in plant growth, development, and stress response. Licorice is a traditional medicinal herb in China; however, abiotic stress has become a critical bottleneck affecting its yield and geographical distribution. In recent years, as the genomes of many plant species have been sequenced, genome-wide characterization of the *TCP* gene family has been carried out in many plant species, including *Gramineae* [35], *Leguminosae* [36], and *Cruciferae* [37]. Studies on the relationship between the *TCP* gene family and abiotic stresses in plants have been reported. TCP transcription factors have been found to interact with a variety of proteins involved in phytohormone signaling pathways, such as MYB and SAP11, during plant development [38,39]. This suggests that TCP transcription factors might play an important role in plant resistance to biotic and abiotic stresses.

In some plants, specific transcription factor families, such as the TCP factor family, may possess redundant genes, or their functions may be supplemented by other transcription factor families, resulting in a smaller number of *TCP* gene family members [40]. During the evolutionary process of plant genomes, events such as gene loss and duplication may occur. Over a long period of evolution, the genome of licorice has undergone a relatively small-scale expansion of *TCP* genes. Compared to some model plants, such as Arabidopsis, licorice may not have experienced large-scale genomic variations like gene expansion, duplication, or inter-species genome-level changes [41]. Licorice is a plant with strong adaptability, typically growing in arid or saline–alkali environments. Members of the *TCP* gene family play an important role in plant morphogenesis, including branching and leaf development [42]. However, licorice may differ from other plants in these developmental mechanisms. Its adaptability mechanisms may rely more on other types of transcription factors or regulatory mechanisms, and the number and function of the *TCP* gene family have not been expanded to the scale seen in other plant species.

In this study, the *TCP* gene family in licorice was analyzed and identified at the whole-genome level, and a total of 24 *TCP* genes were obtained, which were evenly distributed on 24 chromosome scaffolds. By analyzing the physicochemical properties of GiTCP proteins, it was found that all the proteins of this family had good hydrophilicity. Additionally, there were significant differences in the molecular weight and isoelectric point of the proteins (Table 1), which were similar to those of other plant TCP proteins. It is hypothesized that the variability of GiTCP proteins may be related to the involvement of different *GiTCP* genes in different organ development signaling processes [43]. Subcellular localization predictions showed that all 24 *GiTCP* family members were localized to the nucleus, and some were also localized to the cytoplasm, similar to the results reported in *Brassica juncea* [37], *Medicago truncatula* [44], and other species. The secondary structures were mainly α-helical and irregularly coiled.

A phylogenetic analysis showed that the licorice *TCP* gene family is mainly divided into three subfamilies: *PCF*, *CIN,* and *CYC*/*TB1*. In the evolutionary tree, we found that three pairs, *GiTCP14* and *AtTCP2*, *GiTCP3* and *AtTCP11*, and *GiTCP8* and *AtTCP15*, were on the same branch, among which *GiTCP14* may be similar to *AtTCP2* and plays an important role in the regulation of plant leaf morphology and size [45]. *GiTCP3* and *AtTCP11* have similar functions and play an important role in the regulation of vascular bundle development [46]. *GiTCP8* is functionally similar to *AtTCP15*, which can affect cell differentiation and proliferation and the development of organs [47]. The high homology between the *GiTCP* genes and the *MsTCP* gene indicates that they are closely related [10].

Conserved motif and structural domain analyses revealed that all 24 *GiTCP* genes contained motif 1, indicating that this motif was highly conserved throughout the evolution of the TCP gene family in licorice. The results of a gene structure analysis showed that the gene structure of *GiTCPs* was relatively simple, with a small number of introns ranging from 0 to 4, similar to the results of studies on species such as *Solanum Muricatum* [48] and *Elaeis gineensis* Jacq [49].

In addition, the prediction of cis-acting elements in the promoter of the licorice *TCP* gene revealed that the light-responsive element was the most abundant, as well as elements responsive to phytohormones, stress, etc., suggesting that the *TCP* family of genes in plants is involved in many biological processes, such as photosynthesis, growth and developmental regulation, and stress response [50,51,52]. It is hypothesized that the *GiTCP* gene family also plays an important role in growth and development, as well as in the regulation of various stresses.

The results of GO annotation suggest that *GiTCP* genes are involved in a transcriptional regulatory network that plays a role in cell development, differentiation, and response to environmental changes. The present study combined transcriptome and qRT-PCR analyses to illustrate that the *GiTCP* genes were differentially expressed at different times under UV-B radiation. For example, *GiTCP8*, *GiTCP22,* and *GiTCP24* were highly expressed in the roots of licorice; *GiTCP1*, *GiTCP2*, *GiTCP4*, *GiT*CP5, *GiTCP10*, *GiTCP13*, *GiTCP18*, *GiTCP20,* and *GiTCP21* were barely expressed in the roots; and the other *GiTCP* genes showed a low expression. *GiTCP6* and *GiTCP23* peaked after 7 d of UV-B radiation in licorice. Hao et al. reported that many *CrTCP* genes, such as *CrTCP2*, *CrTCP4*, *CrTCP5, CrTCP8*, *CrTCP9*, *CrTCP10*, *CrTCP12*, *CrTCP13*, and *CrTCP15*, were significantly reduced at different time points in periwinkle under UV-B radiation. However, *CrTCP14* expression was significantly induced by radiation with an intensity of 10 μmol (m^2^s)^−1^ after 3 h and significantly decreased after 24 h. The expression of *CrTCP6* was significantly down-regulated at 6 h and up-regulated at 24 h [53]. In *Andrographis paniculata*, the expression levels of all *TCP* genes were down-regulated under UV-B radiation, except for those of *ApTCP2*, *ApTCP5*, *ApTCP6*, *ApTCP15*, *ApTCP18*, and *ApTCP23,* which were up-regulated. In addition, the expression of *ApTCP8*, *ApTCP9*, *ApTCP13*, and *ApTCP17* was not detected [12], further confirming that TCP transcription factors play an important role in response to UV-B radiation. Licorice showed a certain resistance to abiotic stress, which promoted the production of secondary metabolites to a certain extent. Li found that a total of six *GiHDMT* genes (*GiHDMT8*, *11*, *15*, *13*, *21,* and *26*) were down-regulated and that *GiHDMT*23 was up-regulated after NaCl (200 mM) stress [54]. After Na_2_CO_3_ (100 mM) stress, the expression levels of *GiHDMT18*, *GiHDMT13*, *GiHDMT20*, *GiHDMT19,* and *GiHDMT15* were down-regulated, and GiHDMT 23 was up-regulated. After ABA (100 μM) treatment, it was found that only one gene (*GiHDMT1*) was induced, and the expression of three genes (*GiHDMT8, GiHDMT13,* and *GiHDMT20*) was significantly inhibited. After treatment with MeJA (100 μM), the expression levels of all *GiHDMTs* were decreased, but the expression levels of *GiHDMT1*, *GiHDMT11,* and *GiHDMT23* varied non-significantly.

In summary, this study systematically analyzed 24 *GiTCP* gene family members based on bioinformatics and transcriptome data of two varieties of licorice under UV-B radiation, and it preliminarily investigated the biological functions of *GiTCP* genes, thereby providing a theoretical foundation for future in-depth studies on the molecular functions and regulatory roles of *GiTCP* genes in licorice development.

## 4. Materials and Methods

### 4.1. Plant Materials, Growth Conditions, and Treatments

Seeds of *Glycyrrhiza inflata* were obtained from Gansu Provincial Economic Crops Technology Popularization Station (No.282 East Xijin Road, Anning District, Lanzhou City, Gansu Province, China). The species was identified by Professor Pingshun Song of Gansu University of Chinese Medicine. Licorice seeds were selected and soaked in 98% H_2_SO_4_ for 30 min to break seed dormancy, stirred every 10 min, and rinsed with distilled water to remove residual sulfuric acid. They were then sterilized using 3% NaClO (Gansu Elvey Scientific Instrument, Lanzhou, China) for 12 min and submerged in 75% ethanol for 2 min before being rinsed clean using distilled water. The treated seeds were germinated on Petri dishes for 5 d, and uniformly grown seedlings were transplanted to pots containing nutrient soil and vermiculite in a ratio of 5:3, during which time they were watered at 7 d intervals using Hoagland nutrient solution. All seedlings were grown at a temperature of 25 °C in a greenhouse with a light intensity of 500 μmol (m^2^s)^−1^ and a light/darkness cycle of 12/12 h. The seedlings were allowed to grow until they were 90 d old. Licorice plants were selected and then irradiated with UV-B (214 μW·m^−2^) for durations of 0, 7, and 15 d. Their roots were subsequently taken, rinsed with sterilized water, and snap-frozen in liquid nitrogen. The samples were stored in a refrigerator at −80 °C. Three biological replicates were prepared for each treatment group.

### 4.2. Identification of TCP Gene Family Members in Licorice

The *GiTCP* gene family was screened and identified based on the genome sequence database of licorice (http://ngs-data-archive.psc.riken.jp/Gur-genome/download.pl, accessed on 10 January 2024) combined with unpublished transcriptomic data of *G. inflata* obtained from our group [20]. The protein sequences of the *AtTCP* gene family were downloaded from TAIR (https://www.arabidopsis.org/, accessed on 10 January 2024). TBtools (Version number: 2.007) was utilized to BLAST the AtTCP protein sequences with the transcriptome data. Preliminary candidate sequences for the *GiTCP* family members were obtained. The obtained sequences were further screened and confirmed using Pfamscan (https://www.ebi.ac.uk/Tools/pfa/pfamscan/, accessed on 10 January 2024) to eliminate candidate sequences without TCP structural domains (PF03634). Finally, all the TCP family members in licorice were obtained. An analysis of the physicochemical properties of the GiTCP proteins was performed on the website ExPASy (https://web.expasy.org/protparam/, accessed on 11 January 2024). Subcellular localization prediction of the *GiTCP* genes was performed on the website WOLF PSORT (https://wolfpsort.hgc.jp/, accessed on 11 January 2024) [55].

### 4.3. Domain, Gene Structure, and Conserved Motifs of TCP Proteins in Licorice

The GiTCP protein sequences were uploaded to the website MEME (https://meme-suite.org/meme/, accessed on 11 January 2024) to analyze conserved motifs, with the parameter of the corresponding number of motifs set to 10 and the rest of the parameters set to default, and the MAST.XML file was downloaded. The conserved structural domains of the TCP proteins were analyzed using the NCBI CDD tool (https://www.ncbi.nlm.nih.gov/Structure/cdd/wrpsb.cgi, accessed on 11 January 2024). Finally, they were visualized and analyzed using TBtools [56].

### 4.4. Chromosomal Mapping and Secondary Structure Analysis of TCP Genes in Licorice

The physical locations of the *GiTCP* genes were determined on the chromosomes using the gff file of the licorice genome. A physical map of the genes on the chromosomes was constructed using TBtools, as well as an intraspecific collinearity analysis conducted using MCScanX (an add-on of TBtools 2.007) [57]. The secondary structure of the GiTCP proteins was predicted using the online website SOPMA (https://npsa-prabi.ibcp.fr/cgi-bin/npsa_automat.pl?page=npsa_SOPMA.html, accessed on 11 January 2024).

### 4.5. Phylogenetic Tree Analysis of TCP Genes in Licorice

The homostructural domain protein sequences of *G. inflata*, *Arabidopsis thaliana*, *Eucommia ulmoides,* and *Medicago sativa* were used for a phylogenetic analysis. MEGA11 software was used for a sequence comparison analysis, and Neighbor-Joining (NJ) was used to construct a phylogenetic tree of the above four species, with the BootStrap calibration parameter set to 1000, all other parameters set to default, and iTOL (https://itol.embl.de/, accessed on 12 January 2024) used for beautification [58].

### 4.6. Analysis of Cis-Acting Elements of TCP Gene Family in G. inflata

TBtools software was utilized to perform a search for promoter cis-acting elements by extracting a 2000 bp sequence upstream of the start codon of the *GiTCP* gene from the transcriptome data of *G. inflata* and submitting it to the online website PlantCARE (http://bioinformatics.psb.ugent.be/webtools/PlantCARE/html, accessed on 12 January 2024) for a predictive analysis [59].

### 4.7. Construction of TCP Protein Interaction Network in G. inflata

The model plant *Arabidopsis thaliana* was used to predict the interactions of the TCP protein network structures of the homologous genes of licorice and Arabidopsis. Protein network interaction maps were constructed using the online website STRING (https://cn.string-db.org/, accessed on 13 January 2024) [60].

### 4.8. Subcellular Localization Assays

The constructed vector plasmid was transferred into *Agrobacterium tumefaciens* (GV3101) by electrotransformation method and incubated at 30 °C for 2 d. Then, *Agrobacterium tumefaciens* was scraped off a solid Petri dish with an inoculation ring, connected to 10 mL of the corresponding resistant YEB liquid medium, and then incubated at 170 rpm one min for 1 h to collect the bacterial body (4000 rpm one min, centrifuged for 4 min). Then, the supernatant was removed, and the bacteria were resuspended in 10 mM MgCl_2_ (containing 120 μM AS) suspension, with the OD600 adjusted to approximately 0.6. Tobacco plants in good growth conditions were selected and injected into the lower epidermis of the leaves using a 1 mL syringe with the tip removed and labeled well. The injected tobacco plants were cultured in low light for 2 d. The labeled Agrobacterium-injected tobacco leaves were taken, made into slides, observed under a laser confocal microscope, and photographed.

### 4.9. Transcriptome Sequencing Analysis

The total RNA was extracted from the collected licorice root samples using the Trizol reagent kit (Invitrogen, Carlsbad, CA, USA) according to the manufacturer’s protocol. The quality of the RNA was assessed on an Agilent 2100 Bioanalyzer (Agilent Technologies, Palo Alto, CA, USA) and checked using Rnase-free agarose gel electrophoresis (Invitrogen, Carlsbad, CA, USA). After extraction of the total RNA, eukaryotic mRNA was enriched by Oligo (dT) beads. Then, the enriched mRNA was fragmented into short fragments using fragmentation buffer and reverse-transcribed into cDNA by using NEBNext Ultra RNA Library Prep Kit for Illumina (NEB#7530, New England Biolabs, Ipswich, MA, USA). The purified double-stranded cDNA fragments were end-repaired, had a base added, and were ligated to Illumina sequencing adapters. The ligation reaction was purified with the AMPure XP Beads (Invitrogen, Carlsbad, CA, USA) and amplified by polymerase chain reaction (PCR). The cDNA library was sequenced using Illumina Novaseq6000 by Gene Denovo Biotechnology Co. (Guangzhou, China). The transcriptome data of the roots of *G. inflata* under UV-B radiation have been deposited in the National Center of Biotechnology Information Gene Expression Omnibus (NCBI GEO) repository (http://www.ncbi.nlm.nih.gov/geo) under accession number PRJNA1086199.

### 4.10. GO Analysis of TCP Genes in Licorice

Gene functional classification and annotation were conducted using the online software of Gene Denovo Biotechnology Co., Ltd. (https://www.omicshare.com/tools/, accessed on 10 October 2023).

### 4.11. RNA Isolation and qRT-PCR

The CDS sequences of the *GiTCP* genes were identified from the transcriptome data, and primers (Table 4) were designed through NCBI (National Center for Biotechnology Information (nih.gov)). The gene β-actin was selected as the internal reference gene. Total RNA was extracted using TIANGEN’s Plant Tissue Rapid RNA Extraction Kit (Tiangen Biotech, Beijing, China) and then reverse-transcribed into cDNA with a cDNA Synthesis Kit (Tiangen Biotech, Beijing, China) for a qRT-PCR assay, which was repeated three times for each sample. Finally, the relative expression of the *GiTCP* genes was calculated and analyzed using the 2^−ΔΔCt^ algorithm and plotted using Excel 2019, SPSS 23, and Origin 2021 [61,62].

## 5. Conclusions

In this study, 24 *GiTCP* genes were identified in licorice at the genome level, and their structure and function were analyzed using bioinformatics. The *GiTCPs* were divided into Class I and Class II, and they were further divided into three subfamilies (namely, PCF, CYC/TB1, and CIN). In addition, a promoter analysis showed that the GiTCP promoter region usually contained cis-acting elements that respond to abiotic stresses, such as light-responsive, defense-responsive, and stress-responsive elements. A collinearity analysis showed that two pairs of genes had fragment duplication, which played a key role in the expansion of the *GiTCP* gene family. On this basis, the genes with a certain potential resistance to UV-B radiation were identified as candidate genes by comparing them with the transcriptome data of licorice. The expression patterns of the candidate genes were analyzed using qRT-PCR. *GiTCP3*, *GiTCP6*, *GiTCP7*, *GiTCP8*, *GiTCP11*, *GiTCP12*, *GiTCP14*, *GiTCP17*, *GiTCP22*, *GiTCP23,* and *GiTCP24* were found to respond to UV-B radiation. This study lays an important foundation for further research on the function of the *GiTCP* gene family, especially under UV-B radiation. It also provides an important potential application value for the breeding of stress tolerance in licorice.

## Figures and Tables

**Table 1:** Analysis of physical and chemical properties of the *GiTCP* gene family in *G. inflata*.

Gene Name	Sequence ID	AA	MW	pI	II	AI	GRAVY	SL
*GiTCP1*	Glyur000008s00001296.1	433	48,419.6	6.35	56.22	58	−0.688	N,C
*GiTCP2*	Glyur000014s00002559.1	424	46,637.73	6.56	55.14	60	−0.806	N,C,CP
*GiTCP3*	Glyur000020s00001802.1	426	46,357.61	6.39	57.12	53	−0.877	N,C
*GiTCP4*	Glyur000039s00004191.1	378	42,805.57	6.46	55.23	46	−1.098	N,C
*GiTCP5*	Glyur000050s00005694.1	369	40,912.51	8.97	59.19	62	−0.729	N,C,E
*GiTCP6*	Glyur000073s00007830.1	421	45,416.08	7.41	70.02	58	−0.769	N,C
*GiTCP7*	Glyur000175s00012061.1	375	39,457.06	6.26	47.75	62	−0.461	N
*GiTCP8*	Glyur000192s00009341.1	507	54,268.62	7.26	60.82	58	−0.736	N
*GiTCP9*	Glyur000208s00014261.1	357	39,483.73	6.17	66.29	59	−0.815	N
*GiTCP10*	Glyur000275s00017056.1	406	44,812.15	9.32	54.38	67	−0.623	N,C,E
*GiTCP11*	Glyur000278s00017293.1	524	57,436.66	7.42	52.22	61	−0.736	N,CP
*GiTCP12*	Glyur000329s00019843.1	429	46,829.39	6.33	41.64	64	−0.559	N,CP,M
*GiTCP13*	Glyur000418s00021337.1	372	41,089.66	7.13	53.33	69	−0.617	N,CP
*GiTCP14*	Glyur000513s00025388.1	420	43,972.18	6.42	55.38	57	−0.606	N
*GiTCP15*	Glyur000523s00021974.1	429	48,570.05	7.31	51.61	57	−0.943	N,Pm
*GiTCP16*	Glyur000529s00019352.1	380	40,865.72	6.15	53.77	61	−0.611	N,CP,M
*GiTCP17*	Glyur000594s00025967.1	490	54,254.93	6.68	58.91	51	−1.027	N,CP
*GiTCP18*	Glyur000643s00026942.1	392	43,118.88	7.91	52.49	67	−0.754	N,C,CP
*GiTCP19*	Glyur000658s00024843.1	209	22,115.54	8.46	63.38	70	−0.266	N,C,M
*GiTCP20*	Glyur000815s00035011.1	512	57,147.71	9.42	55.92	57	−0.972	N
*GiTCP21*	Glyur001815s00041763.1	378	42,273.69	9.2	56.38	72	−0.598	N,CP
*GiTCP22*	Glyur002854s00044943.1	329	34,959.7	9.01	51.47	66	−0.751	N,CP,E
*GiTCP23*	Glyur002883s00039141.1	343	36,224.26	9.72	62.61	73	−0.334	N,Pm
*GiTCP24*	Glyur007388s00045515.1	256	27,486.88	9.38	54.13	68	−0.627	N,CP,M

Note: AA, amino acid sequence length; MW, molecular weight; pI, isoelectric point; GRAVY, grand average of hydropathicity; II, instability index; AI, aliphatic index; SL, subcellular localization; N, nucleus; C, cytoplasm; CP, chloroplast; E, extracellular matrix; M, mitochondria; Pm, plasma membrane.

**Table 2:** Secondary structure prediction of GiTCP proteins.

Gene Name	Sequence ID	Alpha Helix	Extended Strand	Beta Turn	Random Coil
*GiTCP1*	Glyur000008s00001296.1	27.25%	12.24%	1.85%	58.66%
*GiTCP2*	Glyur000014s00002559.1	19.81%	11.56%	3.77%	64.86%
*GiTCP3*	Glyur000020s00001802.1	15.49%	12.44%	3.99%	68.08%
*GiTCP4*	Glyur000039s00004191.1	33.07%	8.73%	2.38%	55.82%
*GiTCP5*	Glyur000050s00005694.1	15.18%	13.28%	3.25%	68.29%
*GiTCP6*	Glyur000073s00007830.1	20.67%	11.88%	4.75%	62.71%
*GiTCP7*	Glyur000175s00012061.1	12.53%	12.80%	3.47%	71.02%
*GiTCP8*	Glyur000192s00009341.1	19.13%	10.65%	3.35%	66.86%
*GiTCP9*	Glyur000208s00014261.1	14.85%	12.32%	3.08%	69.75%
*GiTCP10*	Glyur000275s00017056.1	18.72%	13.05%	5.42%	62.81%
*GiTCP11*	Glyur000278s00017293.1	28.05%	11.64%	3.63%	56.68%
*GiTCP12*	Glyur000329s00019843.1	22.38%	14.22%	3.03%	60.37%
*GiTCP13*	Glyur000418s00021337.1	18.82%	7.80%	3.49%	69.89%
*GiTCP14*	Glyur000513s00025388.1	22.14%	11.90%	3.57%	62.71%
*GiTCP15*	Glyur000523s00021974.1	36.60%	6.76%	2.33%	54.31%
*GiTCP16*	Glyur000529s00019352.1	14.47%	11.84%	3.42%	70.28%
*GiTCP17*	Glyur000594s00025967.1	25.21%	14.17%	7.5%	53.12%
*GiTCP18*	Glyur000643s00026942.1	13.27%	13.52%	4.59%	68.62%
*GiTCP19*	Glyur000658s00024843.1	19.62%	20.57%	2.87%	56.94%
*GiTCP20*	Glyur000815s00035011.1	20.70%	15.23%	4.10%	59.96%
*GiTCP21*	Glyur001815s00041763.1	38.89%	12.70%	5.29%	43.12%
*GiTCP22*	Glyur002854s00044943.1	23.40%	8.81%	6.08%	61.70%
*GiTCP23*	Glyur002883s00039141.1	20.41%	16.62%	4.66%	58.31%
*GiTCP24*	Glyur007388s00045515.1	25.0%	16.80%	3.12%	55.08%

**Table 3:** GO classification of the annotated *GiTCP* genes in *G. inflata*.

Class	GO Term	Annotation	*GiTCP* Genes
MF	GO:0005488	Binding	*GiTCP1*, *GiTCP14*, *GiTCP16*, *GiTCP19*, *GiTCP23*
	GO:0140110	Transcription regulator activity	*GiTCP14*, *GiTCP16*, *GiTCP19*, *GiTCP23*, *GiTCP24*
CC	GO:0032991	Protein-containing complex	*GiTCP24*
	GO:0110165	Cellular anatomical entity	*GiTCP14*, *GiTCP16*, *GiTCP19*, *GiTCP23*, *GiTCP24*
BP	GO:0000003	Reproduction	*GiTCP14*
	GO:0008152	Metabolic process	*GiTCP1*, *GiTCP14*, *GiTCP16*, *GiTCP19*, *GiTCP23*, *GiTCP24*
	GO:0009987	Cellular process	*GiTCP1*, *GiTCP14*, *GiTCP16*, *GiTCP19*, *GiTCP23*, *GiTCP24*
	GO:0022414	Reproductive process	*GiTCP14*
	GO:0032501	Multicellular organismal process	*GiTCP14*, *GiTCP19*, *GiTCP23*
	GO:0032502	Developmental process	*GiTCP14*, *GiTCP19*, *GiTCP23*
	GO:0048511	Rhythmic process	*GiTCP14*, *GiTCP19*
	GO:0048519	Negative regulation of biological process	*GiTCP23*
	GO:0050789	Regulation of biological process	*GiTCP14*, *GiTCP16*, *GiTCP19*, *GiTCP23*
	GO:0050896	Response to stimulus	*GiTCP14*, *GiTCP16*
	GO:0065007	Biological regulation	*GiTCP14*, *GiTCP16*, *GiTCP19*, *GiTCP23*

**Table 4:** qRT-PCR primer sequences.

**Gene Name**	**Forward Primer (5′-3′)**	**Reverse Primer (5′-3′)**
*GiTCP3*	CAAACTCCTCGTCAACCCGA	CTTTGTGTGCCGGTCCTTTG
*GiTCP6*	CCGAATTAGCCGCGAACAAG	GAGCTCACGTGTCAGTTGGA
*GiTCP7*	GCAGCGACGAGTATTCCAGA	CGCTGGAACTAACACTGGGT
*GiTCP8*	TCTACCAGCCCTCTCAGCAT	GATCGGAATTGTCGGTGGGT
*GiTCP11*	CGTGTCTGGCCTTTTCCGAT	CCCAAATGGTCTTTCCTTGCG
*GiTCP12*	GCTCCTGTTGGGTTTGATGC	AGGGTCCCCCTATGGGAAAA
*GiTCP14*	GCGAGACGATAGAGTGGCTC	CGGAGAAGGTGCTAGGGTTG
*GiTCP17*	GGCACCACCACTCATCAAGA	GCTGTTGTCACTGAGAGCCT
*GiTCP22*	CCTGGTTTGGAACTGGGGTT	GGCCATGCAATTCCACCTTG
*GiTCP23*	TGTTCAGCTCCAACCAGCAA	TGGAAGAAGAGCGCGACAAT
*GiTCP24*	TACCCTCGCGGTGAAGAAAC	GCGGCGATAATTGACGGTTC
*β-actin*	CCTCATGCCATCCTTCGTC	TCTTTGCAGTCTCGAGTTCTTG

## Data Availability

The data are available in the National Center of Biotechnology Information Gene Expression Omnibus (NCBI GEO) repository (http://www.ncbi.nlm.nih.gov/geo) under accession number of PRJNA1086199.

## Supplementary Materials

The following supporting information can be downloaded at: https://www.mdpi.com/article/10.3390/ijms26010159/s1.

## Abbreviations

TCP	teosinte branched 1/cincinnata/proliferating cell factor
TB1	teosinte branched 1
PCF	proliferating cell factor
CYC	cycloidea
Ms	Medicago sativa L
Ma	Musa acuminata
Pg	Panax ginseng
Ap	Andrographis paniculata
Dch	Dendrobium
Gi	*G. inflata* Bat
GA3	gibberellic acid 3
MeJA	methyl jasmonate
bZIP	basic region/leucine zipper
AA	amino acid sequence length
MW	molecular weight
pI	isoelectric point
GRAVY	grand average of hydropathicity
II	instability index
AI	aliphatic index
SL	subcellular localization
N	nucleus
C	cytoplasm
CP	chloroplast
E	extracellular matrix
M	mitochondria
Pm	plasma membrane
At	Arabidopsis thaliana
Eu	Eucommia ulmoides
GO	gene ontology
qRT-PCR	quantitative real-time PCR
UV-B	ultraviolet B
Cr	Catharanthus roseus
